# Impairments in quality of life and predictors of symptom burden in patients with hypoparathyroidism: results from a population-based survey

**DOI:** 10.1007/s12020-023-03443-2

**Published:** 2023-07-14

**Authors:** Matthias Büttner, Dieter Krogh, Heide Siggelkow, Susanne Singer

**Affiliations:** 1 Institute of Medical Biostatistics, Epidemiology and Informatics (IMBEI), University Medical Center Mainz, Mainz, Germany; 2 University Cancer Centre, Mainz, Germany; 3 Netzwerk Hypopara im Bundesverband Schilddrüsenkrebs—Ohne Schilddrüse leben e.V, Berlin, Germany; 4 Clinic of Gastroenterology, Gastrointestinal Oncology and Endocrinology, University of Göttingen, Göttingen, Germany; 5 MVZ Endokrinologikum Göttingen, Göttingen, Germany

**Keywords:** Hypoparathyroidism, Quality of life, Symptom burden, HPQ28

## Abstract

**Purpose:**

To investigate the quality of life (QoL) in patients with hypoparathyroidism (hypoPT) compared to the general population and to identify sociodemographic and clinical factors that are associated with symptom burden.

**Methods:**

Patients with a diagnosis of hypoPT participated in an online survey. Information regarding the survey was distributed by treating physicians or a self-help organization. Quality of life was assessed using the EORTC QLQ-C30 and symptom burden using the Hypoparathyroid Patient Questionnaire (HPQ28). Multivariate linear regression analysis was used to compare QoL of hypoPT patients with the general population (adjusted for age, sex, education)and to identify factors associated with symptom burden.

**Results:**

Altogether, 264 hypoPT patients provided information on QoL and symptom burden. HypoPT was associated with worse *cognitive* (β = −15.9; *p* < 0.01) and *emotional functioning* (β = −12.3; *p* = 0.04) compared to the general population. The highest symptom burden in hypoPT patients was observed for the domains *loss of vitality* (mean: 61.4; SD: 21.9), *pain and cramps* (mean: 43.7; SD: 26.5), and *numbness and tingling sensations* (mean: 38.9; SD: 30.0). Female gender was associated with a higher symptom burden across all nine domains of the HPQ28, while longer disease duration was associated with a lower symptom burden in *neurovegetative symptoms*, *loss of vitality*, *depression and anxiety*, and *depressive symptoms*.

**Conclusion:**

HypoPT patients have impaired QoL compared to the general population. Being female is strongly associated with high symptom burden.

## Introduction

With prevalence numbers in different countries ranging from 9.4 to 37 per 100,000, hypoparathyroidsm (hypoPT) is a rare endocrine disorder [1–4]. Chronic hypoPT is defined by hypocalcaemia with inappropriately normal or low parathyroid hormone for more than six months. HypoPT is the only endocrine disease where the missing hormone cannot be replaced; thus, the standard therapy is calcium and vitamin D supplementation [5, 6]. During the last years, new treatment options like synthetic PTH analog (PTH 1-34) and human recombinant parathormone (PTH 1-84) have been introduced for patients whose hypocalcemia cannot be treated with the standard treatment [7–10].

Studies have shown that patients with hypoPT have impairments in quality of life (QoL) compared to the general population or matched controls [1, 11–13]. A high frequency of hypoPT symptoms is also reflected in poorer QoL in hypoPT patients [14]. Symptoms of hypoPT cover physical (e.g. fatigue, paresthesias, muscle cramps), cognitive (e.g. brain fog, memory loss), and emotional (e.g. anxiety, depression) domains [15, 16]. Often studies focusing on symptom burden have investigated the association between blood level concentrations (e.g. serum calcium) and symptom burden. Results are conflicting, with some studies observing an association between symptom burden and blood level concentrations [17, 18] and others not reporting this association [1, 13, 19]. Since blood concentrations levels might vary throughout the day, single measures might not fully explain the impact on symptom burden [10]. Sociodemographic (e.g. age, sex) and clinical (e.g. cause of hypoPT, time since diagnosis) factors and their impact on symptom burden have not so often been investigated. Additionally, symptoms have rarely been assessed with validated questionnaires.

The aim of this study was to investigate the QoL in hypoPT patients and compare it to the general population while adjusting for sociodemographic factors. Additionally, this study aims at reporting the symptom burden of hypoPT and to identify clinical and sociodemographic factors which might be associated with symptom burden.

## Methods

### Design

An online survey for patients with hypoPT was created and made available from 10/2020 to 10/2021. Participants were informed about the survey through the patient organization “Netzwerk Hypopara”, and by their treating physician, who were contacted by the study coordinators. Additionally, the online survey was supported by the German Society of Endocrinology and the German Society of Nuclear Medicine. A paper-based version was available upon request, and the data was then entered into the database by the study center. Details regarding the study, its design, and detailed information on the assessments can be found elsewhere [14].

A confirmed diagnosis of hypoPT by the treating physician that was at least six months in the past and age above 18 years were the eligibility criteria. As per local regulations, no ethics committee approval was needed due to the anonymity of the data (confirmed by the Ethics Committee of the Landesärztekammer Rhineland-Palatinate).

Raw data for the German general population of the European Organization for Research and Treatment of Cancer (EORTC) Quality of Life Core Questionnaire (QLQ-C30) was obtained via the EORTC Quality of Life Group from a study by Nolte et al. [20].

### Assessments

Clinical and sociodemographic data were obtained through the patients themselves. Quality of life was assessed using the functioning scales (*physical functioning* (PF), *role functioning* (RF), *emotional functioning* (EF), *cognitive functioning* (CF), *social functioning* (SF)) and the *global quality of life* (QL) of the EORTC QLQ-C30 [21]. Response formats of the respective items are presented on a four-point Likert scale (“not at all”, “a little”, “quite a bit”, and “a lot”).

HypoPT symptoms were assessed by the Hypoparathyroid Patient Questionnaire HPQ28 [16]. The HPQ28 consists of six multi-item scales (*pain and cramps* (PaC), *loss of vitality* (LoV), *neurovegetative symptoms* (NVS), *gastrointestinal symptoms* (GiS), *depression and anxiety* (DaA), and *depressive symptoms* (PHQ)) and three single item scales (*numbness and tingling sensations* (NaTS), *rapid heartbeat* (RH), and *memory problems* (TM)). Items are assessed on a four-point Likert scale (0 = “not at all,” 1 = “slightly,” 2 = “moderately,” 3 = “severely/strongly”) using a four-week timeframe. Scales range from 0 to 100 with higher scores indicating a higher symptom burden.

### Statistical analysis

Scores for symptom burden using the HPQ28 and for the functioning scales of the EORTC QLQ-C30 were calculated according to their scoring guidelines [16, 22]. Population (for the hypoPt and general population) characteristics are presented using mean values or percentages according to the data. Univariate comparisons between the two groups (hypoPT and general population) for population characteristics and QoL scores were performed using chi-square-tests, Mann-Whitney-U tests, or *t* tests depending on the distribution of the data. For QoL, multivariate linear regression models with the independent variables age, sex, education and group (hypoPT vs. general population) with all possible interaction terms were used. For the identification of factors which might be associated with symptom burden in hypoPT patients, multivariate linear regression models with age, sex, time since diagnosis (0.5-2years vs. 2–5 years vs. more than five years), treatment (conventional treatment vs. PTH replacement), cause of hypoPT (non-surgical vs. surgical (thyroid cancer) vs. surgical (other reason)), and timing of monitoring of serum calcium levels (at least every six months vs. more than every six months) as independent variables, were performed for all symptom scales. All assumptions for multivariate linear regression were checked by graphical inspection of the relevant plots. All statistical analyses were performed using R (version 4.0.4, R Foundation for statistical computing).

## Results

### Sample and population characteristics

In total, 268 participants provided data, with 264 (98.5%) being eligible for analysis. Since their hypoPT diagnosis was less than six months ago and therefore it could not be excluded that their hypoPT is transient, four (1.5%) participants were excluded from the analysis.

General population data was available from 1006 individuals. Characteristics of both populations are presented in Table 1. In the hypoPT population, a significantly higher share of the participants was female (85.2% vs. 49.8%; *p* < 0.01), but no significant differences for age or education were observed.

**Table 1:** Patient and general population characteristics using mean (SD) or n (%)

	HypoPT (*n* = 264)	General population (*n* = 1006)	*p* value
Characteristic			
Age (mean (SD))	54.5 (13.3)	53.8 (15.0)	0.48
Sex (n (%))			<0.01
male	36 (13.6)	505 (50.2)	
female	225 (85.2)	501 (49.8)	
missing	3 (1.1)	0	
Education (n (%))			0.57
below 10 years	36 (13.6)	113 (11.2)	
10 years	102 (38.6)	396 (39.4)	
more than ten years	125 (47.3)	489 (48.6)	
missing	1 (0.4)	8 (0.8)	
Living situation (n (%))		NA	
alone	61 (23.1)		
with someone	203 (76.9)		
Occupation (n (%))		NA	
employed/self-employed	145 (54.9)		
regular retirement	57 (21.6)		
unable to work	45 (17.0)		
other	17 (6.4)		
Member of Self-help organization (n (%))		NA	
no	167 (63.3)		
yes	97 (36.7)		
Time since diagnosis (n (%))		NA	
6 months - 1 year	6 (2.3)		
1–2 years	22 (8.3)		
2–5 years	47 (17.8)		
5–10 years	43 (16.3)		
more than 10 years	145 (54.9)		
missing	1 (0.4)		
Cause of HPT (n (%))		NA	
non-surgical	21 (8.0)		
* genetic/ autoimmune*	10		
* idiopathic*	6		
* other*	5		
surgical	243 (92.0)		
* thyroid cancer*	100 (41.2)		
* adenoma/goiter/nodules*	124 (51.0)		
* hyperparathyroidism*	6 (2.5)		
* hyperthyroidism*	10 (4.1)		
* other*	3 (1.2)		
Symptom burden (n (%))		NA	
none to low	59 (22.3)		
medium	75 (28.4)		
high	130 (49.2)		
Medication (n (%))		NA	
Calcium	153 (58.0)		
Calcitriol	152 (57.6)		
Alphacalcidol	23 (8.7)		
Colecalciferol	66 (25.0)		
PTH	28 (10.6)		
Magnesium	49 (18.6)		
Dihydrotachysterol	31 (11.7)		
missing	13 (4.9)		

### Quality of life in hypoparathyroid patients compared to the general population

Patients with hypoPT had significantly worse QoL scores across all functioning domains compared to the general population (Table 2), with the largest difference seen for CF (56.9 vs. 85.4; *p* < 0.01) and EF (46.9 vs. 75.1; *p* < 0.01). In multivariate analysis, controlling for age, sex, and education and interaction terms, statistically significant effects between the groups (patients vs. general population) were seen for CF (β = −15.9; *p* < 0.01) and EF (β = −12.3; *p* = 0.04). No statistically significant differences were seen for PF (β = −2.0; p = 0.7), RF (β = −9.9; p = 0.2), SF (β = −10.8; *p* = 0.1), and QL (β = 0.9; *p* = 0.9). Significant interactions between sex and group were observed for EF, SF, CF, and QL. No other significant interactions were observed. Higher education was statistically significantly associated with higher QoL scores (except for CF) across all functioning domains of the EORTC QLQ-C30 (Table 3).

**Table 2:** Quality of life scores in patients with hypoparathyroidism (HPT) and in the general population

	All (*n* = 264)	general-population (*n* = 1006)	*p* value
Quality of life (EORTC QLQ-C30)			
Physical functioning (PF)	74.0 (21.5)	82.0 (21.5)	<0.01
Role functioning (RF)	63.6 (32.5)	80.3 (27.4)	<0.01
Social functioning (SF)	61.7 (33.4)	85.1 (25.5)	<0.01
Emotional functioning (EF)	46.9 (30.1)	75.1 (24.2)	<0.01
Cognitive functioning (CF)	56.9 (31.6)	85.4 (21.1)	<0.01
Global Quality of Life (QL)	55.6 (21.3)	65.9 (22.2)	<0.01
Symptoms (HPQ28)			
Pain and Cramps (PaC)	43.7 (26.5)		
Neurovegetative Symptoms (NVS)	28.3 (21.0)		
Loss of Vitality (LoV)	61.4 (21.9)		
Depression and Anxiety (DaA)	32.2 (26.1)		
Gastrointestinal Symptoms (GiS)	23.5 (25.7)		
Trouble Memory(TM)	31.7 (28.9)		
Numbness and Tingling Sensations (NaTS)	38.9 (30.0)		
Rapid Heartbeat (RH)	30.9 (28.9)		
Depressive Symptoms (PHQ)	31.2 (26.7)		

Scores are presented as mean and standard deviation (SD); Univariate comparison using *t* tests

**Table 3:** Quality of life in patients with hypoPT compared with a general population adjusted for age, sex, and education – multivariate linear regression

	PF	p	RF	p	EF	p	SF	p	CF	p	QL	p
Group (ref: norm population)												
HypoPT	−2.0	0.7	−9.9	0.2	−12.3	0.04	−10.8	0.1	−15.9	<0.01	0.9	0.9
Interaction Sex*hypoPT	−6.6	0.1	−9.3	0.09	−12.8	<0.01	−17.1	<0.01	−15.0	<0.01	−9.9	0.02
Age (continuous)	−0.3	<0.01	−0.3	<0.01	0.3	<0.01	0.1	0.6	0.1	0.06	−0.1	<0.01
Sex (ref: male)												
female	−3.7	0.01	−3.8	0.03	−1.3	0.4	0.5	0.8	0.6	0.7	−2.2	0.1
Education (ref: below ten years)												
ten years	7.4	<0.01	7.1	0.02	7.2	<0.01	6.7	0.02	4.0	0.1	6.8	<0.01
more than ten years	8.8	<0.01	8.9	<0.01	9.5	<0.01	7.8	<0.01	5.7	0.02	10.2	<0.01

No statistical significant association for all other interaction terms was observed

### Symptom burden and factors associated with it in patients with hypoparathyroidism

The highest symptom burden in patients with hypoPT was seen for *loss of vitality* (mean: 61.4; SD: 21.9), *pain and cramps* (mean: 43.7; SD: 26.5), and *numbness and tingling sensations* (mean: 38.9; SD: 30.0). An overview of the symptom burden of hypoPT patients is presented in Table 2. In multivariate regression analysis, being female was statistically significantly associated with a higher symptom burden in *pain and cramps* (β = 16.0; *p* < 0.01), *neurovegetative symptoms* (β = 15.8; *p* < 0.01), *loss of vitality* (β = 15.4; *p* < 0.01), *depression and anxiety* (β = 14.5; *p* = 0.04), *gastrointestinal symptoms* (β = 14.4; *p* < 0.01), *memory problems* (β = 12.4; *p* = 0.03), *numbness and tingling sensations* (β = 11.4; *p* = 0.03), *rapid heartbeat* (β = 18.8; *p* < 0.01), and *depressive symptoms* (β = 18.2; *p* < 0.01). Patients 2–5 years post diagnosis (compared to 0.5–2 years) had a statistically significantly lower symptom burden in *depressive symptoms* (β = −20.7; p < 0.01), and patients more than 5 years post diagnosis had lower scores in *neurovegetative symptoms* (β = −11.1; p < 0.01), *loss of vitality* (β = −9.6; *p* < 0.01), *depression and anxiety* (β = −13.8; *p* < 0.01), *rapid heartbeat* (β = −12.2; *p* < 0.01), and *depressive symptoms* (β = −23.7; *p* < 0.01). Patients on PTH replacement therapy (compared to conventional treatment) reported higher symptom scores for *memory problems* (β = 12.2; *p* = 0.04). Monitoring serum calcium levels less frequently than every six months (compared to at least every six months) was associated with a lower symptom burden in *numbness and tingling sensations* (β = −10.4; *p* = 0.01). Age and cause of hypoPT were not statistically significantly associated with symptom burden (Table 4).

**Table 4:** Factors associated with symptom burden in patients with hypoparathyroidism

	PaC	p	NVS	p	LoV	p	DaA	p	GiS	p	TM	p	NaTS	p	RH	p	PHQ	p
Age (continuous)	0.1	0.38	−0.1	0.33	0.3	0.01	−0.2	0.09	−0.2	0.1	−0.1	0.33	−0.2	0.18	0.1	0.84	0.2	0.22
Sex (ref: male)																		
female	16.0	<0.01	15.8	<0.01	15.4	<0.01	14.5	0.04	14.4	<0.01	12.4	0.03	11.4	0.03	18.8	<0.01	18.2	<0.01
Time since diagnosis (ref: 0.5–2 years)																		
2–5 years	4.9	0.43	−4.0	0.41	−4.2	0.43	−11.9	0.07	−6.4	0.32	−7.4	0.30	9.0	0.19	−5.1	0.48	−20.7	<0.01
more than 5 years	−2.0	0.72	−11.1	0.01	−9.6	0.04	−13.8	0.02	−4.5	0.42	−7.9	0.21	−9.4	0.12	−12.2	0.05	−23.7	<0.01
Treatment (ref: conventional treatment)																		
PTH replacement	7.5	0.15	7.0	0.09	6.4	0.15	−0.5	0.93	3.8	0.47	12.2	0.04	8.6	0.13	3.7	0.53	4.9	0.36
Cause of hypoPT (ref: non-surgical)																		
surgical—thyroid cancer	8.8	0.16	−1.3	0.79	5.1	0.36	2.8	0.67	−6.5	0.31	8.0	0.27	13.2	0.06	10.7	0.14	5.8	0.37
surgical—other reason	12.6	0.05	0.1	0.98	3.6	0.51	3.5	0.59	−3.8	0.55	4.7	0.52	11.1	0.11	10.2	0.16	5.5	0.39
Monitoring of Ca-levels (at least every 6 months)																		
More than every 6 months	−5.9	0.12	−5.9	0.05	−0.3	0.92	4.0	0.31	4.2	0.28	2.8	0.53	−10.4	0.01	2.0	0.65	7.0	0.07

*PaC* pain and cramps, *NVS* neurovegetative symptoms, *LoV* loss of vitality, *DaA* depression and anxiety, *GiS* gastrointestinal symptoms, *TM* memory problems, *NaTS* numbness and tingling sensations, *RH* rapid heartbeat, *PHQ* depressive symptoms

## Discussion

The results from our study show that patients with hypoparathyroidism report poorer quality of life than the general population. We also found that patients with hypoparathyroidism report a high number of symptoms.

The pronounced impairments in CF and EF in patients with hypoPT as seen in our study, are in line with findings from other studies. [1, 11, 23, 24]. Hadker et al. [15] reported in a study with 374 hypoPT patients that more than half of the patients described symptoms such as *brain fog/mental lethargy* (72%), inability to focus/concentrate (65%), *memory loss/ forgetfulness* (62%), *anxiety/fear/inner unrest* (59%) and *feeling sad/down/blue/depressed* (53%). In our study symptoms like *neurovegetative symptoms*, *memory problems*, *depression and anxiety* and *depressive symptoms* are present but are not the most dominant symptoms of patients. The cognitive and emotional impairments might be explained by the effect of PTH itself on neurocognitive function. Studies have shown that PTH can activate PTH2 receptors [25], which have been linked to areas in the brain that are relevant for anxiety and depression in animal studies [26, 27]. While other studies [1, 17] also found worse QoL in physical and general QOL domains compared to the general population or matched controls, this was not present in our study. This is surprising since physical symptoms like loss of vitality, or pain and cramps are the most prevalent symptoms in our group of patients, and PTH receptors are also expressed in muscle cells [25, 27–29]. There are possible explanations for these findings. First, compared to others studies [1, 11, 13, 17] we were able to control for education and to evaluate its association on QOL in our study population. In our study, higher education was statistically significantly associated with higher scores across all QOL domains (except for CF in the ten years of education group; see Table 2), indicating a higher QOL in groups with higher education. There are various potential explanation for this finding. Studies have shown that higher education lowers the barriers to use cancer support or information resources [30]. Another possible explanation is that patients with higher education have a better health literacy, are able mobilize support resources and are better at developing coping strategies [31]. Secondly, more than half (55%) of our hypoPT were diagnosed more than ten years ago. They may have adopted or developed coping strategies to deal with their physical symptoms, while for cognitive or emotional problems this might not be easy and may require professional help. In our study 18% of the patients had professional psychological support. Patients who required psychological support had statistical significantly lower scores in CF (37.9 vs. 61.1; p < 0.01) and EF (26.5 vs. 51.3; p < 0.01) compared to patients who had no psychological support.

Over 75% (77.7%) of the participants in our study reported a medium or high symptom burden, with loss of vitality, pain and cramps and numbness and tingling sensations being the most pronounced. The high symptom burden is in line with findings from other studies [15, 32, 33]. Hadker et al. [15] reported that 72% of their study participants reported more than ten different symptoms within the last twelve months, and in Siggelkow et al. [33] 73% of the patients reported one or more moderate, severe or very severe symptom within a seven-day recall period. Compared to emotional or cognitive symptoms, physical symptoms were more pronounced in our study population. This is also in line with findings from other studies [33–35], while patients in Hadker et al. [15] reported an equal share of physical and cognitive symptoms. This may be due to the fact that Hadker et al. [15] used a twelve-month recall period, while this was much shorter in our study and other studies. Interestingly findings from the HPQ28 seem contrary to the findings from our QOL results where the lowest scores were reported for emotional and cognitive functioning. There might be several explanations for this. As described above, it is possible that hypoPT patients adopted to their physical symptoms and developed coping strategies, while this might not be easy for emotional or cognitive impairments. Secondly physical symptoms may also exist through comorbidities or are present in non-hypoPT or general populations. We were not able to assess comorbidities, so we cannot assess this for our population. Büttner et al. [24] investigated symptoms in a population of patients after thyroid cancer with and without hypoPT. In both groups in this study, a high share of patients reported symptoms like *pain in the joints* (hypoPT: 82% vs. no hypoPT: 54%), *attack of tiredness* (hypoPT: 77% vs. no hypoPT: 51%), or *muscle cramps* (hypoPT: 59% vs. no hypoPT: 38%) within the last week. In general, the high symptom burden of hypoPT patients might be explained by several factors. First, PTH receptors have been found in several muscle, spinal cord, and brain regions [25–27, 29]. A PTH deficiency in these regions might contribute to the development of symptoms. Secondly, symptoms might be induced by low levels of blood levels like calcium. In our study we were not able to link blood levels to symptoms due to the fact that the last assessment of blood levels had happened weeks or months before the symptom assessment. The impact of blood levels on QOL and symptoms has been discussed controversially in the literature. While some studies found an association between calcium levels and QOL or symptoms [17, 18], this correlation was not seen in other studies [1, 13, 19].

We aimed at identifying factors which are associated with symptom burden. Being female was associated with a higher symptom burden in all nine symptom scales. This association has been observed in other hypoPT studies [4, 17] and other diseases [36–38]. With the data obtained in our study, we are not able to give an explanation for this association. However, one possible explanation for this is that women find it easier to talk about emotional and social problems than men [39]. Another possible explanation might be the effect of estrogen levels on bone metabolism [40]. Varying estrogen levels during the menstrual cycle might have an impact on calcium levels and therefore might impact QOL.

A longer time since diagnosis was associated with lower symptom scores in *depressive symptoms* (2–5 years since diagnosis compared to 0–5 to 2 years) and *neurovegetative symptoms*, *loss of vitality*, *depression and anxiety*, *rapid heartbeat* and *depressive symptoms* (more than five years since diagnosis compared to 0–5 to 2 years). This effect was not observed in other studies [11, 23]. Possible explanations for our findings may be that patients with longer disease duration are on a stable dosing regimen which has been established over several years while for patients with a shorter disease duration the best dosing regimen still needs to be established. Also patients with longer disease duration may have developed coping strategies to handle their disease and the respective symptoms. Regression analysis also showed an association between regular (at least every six months) monitoring of serum calcium levels and a lower symptom burden in *numbness and tingling sensations*. To our knowledge no study has investigated regular monitoring of serum calcium levels and symptoms. With regular monitoring of calcium levels, the treating physician has the opportunity to adapt the current treatment to the calcium levels and therefore might reduce the risk of hypocalcemia symptoms.

Our study has several limitations. Firstly, through the cross sectional setting we are not able to identify causal relationships. Nevertheless, this is still one of the largest studies investigating QOL and symptom burden in hypoPT Patients. Secondly, no control group was available for the symptom burden. Data from the general population for the HPQ28 has been assessed in another project but is not yet published. Thirdly, we do not have blood levels on the date the patients filled out the questionnaire. Therefore, we are not able to check for a relationship between blood level and QOL or symptoms. However, the effects of current blood levels on QOL and symptoms are controversially discussed in the literature [1, 10, 13, 18, 19]. We do have information on current medications for hypoPT but this information is not detailed enough (e.g. dosing levels, compliance with treatment) to perform in debt analysis. Additionally, we do not have information on co-morbidities of our patients which might have an effect on QOL and symptoms. Our study was performed during the COVID pandemic and therefore this may have had an impact on patients’ QOL or symptom burden, but this was only stated by very few patients in any of the free text options. For the free text option of the QOL section only 7 (2.7%) stated that the COVID pandemic might have an impact on their QOL while for the free text section for the whole questionnaire one (0.4%) participant stated that his answers might be influenced by the pandemic. Therefore, we are quite sure, that the participants provided information on QOL which is not biased by the pandemic. Lastly, one limitation might be the EORTC QLQ-C30 is a questionnaire developed for cancer patients, but it is also used in various non-cancer populations [20] and shows good correlations in the functioning scales of generic QOL questionnaires in non-cancer population [41]. But by using the EORTC QLQ-C30 we were able to compare the QOL to a general population sample which included sex, age and education. Especially education was an important variable for the comparison as very few studies have included this when comparing hypoPT patients QOL to the general population and as our study has shown education has an impact on patients QOL. Another strength of our study was that the assessment of symptoms was done using a validated symptom questionnaire for hypoPT patients, the HPQ28 [16]. Furthermore, our sample size is large and our study population is heterogeneous, making our sample generalizable for hypoPT.

## Conclusion

Patients with hypoPT have a reduced QOL compared to the general population and suffer from various symptoms. Longer disease duration is associated with lower symptom burden. Investigations on potential coping strategies might give an explanation on these findings and might also help other hypoPT patients deal with their disease. Female gender seems to be associated with a higher symptom burden.
